# Bimodal distribution of tone-matching deficits indicates discrete pathophysiological entities within the syndrome of schizophrenia

**DOI:** 10.1038/s41398-019-0557-8

**Published:** 2019-09-06

**Authors:** Clément Dondé, Antigona Martínez, Joshua T. Kantrowitz, Gail Silipo, Elisa C. Dias, Gaurav H. Patel, Juan Sanchez-Peña, Cheryl M. Corcoran, Alice Medalia, Alice Saperstein, Blair Vail, Daniel C. Javitt

**Affiliations:** 1 INSERM, U1028; CNRS, UMR5292; Lyon Neuroscience Research Center, Psychiatric Disorders: from Resistance to Response Team, Lyon, F-69000 France; 2 University Lyon 1, Villeurbanne, F-69000 France; 3 Centre Hospitalier Le Vinatier, Bron, France; 4 Nathan Kline Institute, Orangeburg, NY USA; 5 Deppartment of Psychiatry, Columbia University Medical Center/New York State Psychiatric Institute, New York, NY USA; 6 Department of Psychiatry, Icahn School of Medicine at Mount Sinai, New York, NY USA

**Keywords:** Long-term memory, Diagnostic markers

## Abstract

To date, no measures are available that permit differentiation of discrete, clinically distinct subtypes of schizophrenia (SZ) with potential differential underlying pathophysiologies. Over recent years, there has been increasing recognition that SZ is heterogeneously associated with deficits in early auditory processing (EAP), as demonstrated using clinically applicable tasks such as tone-matching task (TMT). Here, we pooled TMT performances across 310 SZ individuals and 219 healthy controls (HC), along with clinical, cognitive, and resting-state functional-connectivity MRI (rsFC-MRI) measures. In addition, TMT was measured in a group of 24 patients at symptomatic clinical high risk (CHR) for SZ and 24 age-matched HC (age range 7–27 years). We provide the first demonstration that the EAP deficits are bimodally distributed across SZ subjects (*P* < 0.0001 vs. unimodal distribution), with one group showing entirely unimpaired TMT performance (SZ-EAP+), and a second showing an extremely large TMT impairment (SZ-EAP−), relative to both controls (*d* = 2.1) and SZ-EAP+ patients (*d* = 3.4). The SZ-EAP− group predominated among samples drawn from inpatient sites, showed higher levels of cognitive symptoms (PANSS), worse social cognition and a differential deficit in neurocognition (MATRICS battery), and reduced functional capacity. rsFC-MRI analyses showed significant reduction in SZ-EAP− relative to controls between subcortical and cortical auditory regions. As opposed to SZ, CHR patients showed intact EAP function. In HC age-matched to CHR, EAP ability was shown to increase across the age range of vulnerability preceding SZ onset. These results indicate that EAP measure segregates between discrete SZ subgroups. As TMT can be readily implemented within routine clinical settings, its use may be critical to account for the heterogeneity of clinical outcomes currently observed across SZ patients, as well as for pre-clinical detection and efficacious treatment selection.

## Introduction

In 1911, Bleuler famously described the syndrome of *dementia pracecox* as including a “group of schizophrenias” (SZ), reflecting the strong heterogeneity of presentation across individuals^1^. Nevertheless, to date attempts to identify variables that divide individuals with SZ into discrete, dichotomously distributed subtypes based upon symptoms have largely failed. For example, although individuals with SZ show consistent deficits in neurocognitive functioning, such deficits are unimodally distributed across large populations, and so do not yield dichotomous subtypes^2^. Similarly, symptoms of SZ are both unimodally distributed and unstable over time, leading to recent abandonment of clinical subtyping in DSM-5^3^.

Early auditory processing (EAP) deficits in SZ were first demonstrated in 1973^4^ but were not studied systematically until ~20 years ago. Since then EAP deficits have been demonstrated behaviorally using paradigms such as simple tone-matching task (TMT)^5–7^, and neurophysiologically, using measures such as mismatch negativity (MMN)^8–10^. For both types of measures, effect sizes are commensurate with those of more general neurocognitive measures^9^, and contribute highly to impaired functional capacity^7,11^.

Moreover, EAP deficits may be heterogeneously distributed across different samples of patients. For example, in one early study of EAP, deficits in performance were noted primarily in patients drawn from long-term residential care vs. outpatient settings, despite similar illness duration, medication dosage and symptom levels between groups. As compared with their differential deficits in EAP, the groups showed similar susceptibility to same-modality distraction, suggesting relatively similar cognitive control abilities^12^.

Significant heterogeneity of deficit was also observed in a recent meta-analysis of EAP studies (Q(9) = 17.22, *p* = 0.04, *I*^2^ = 47.74%), suggesting likely cohort effects^6^. MMN—a neurophysiological index of EAP dysfunction—is also differentially reduced in patients drawn from primarily inpatient vs. primarily outpatient settings^10^. Here, we pooled measures of TMT across several studies and recruitment sites in order to evaluate the distribution of EAP deficits within a large sample of SZ and HC participants (*n* = 310/219), as well as the relationship of these deficits to symptoms and neuropsychological domains.

It has recently been suggested that the clinical diagnosis of SZ encompasses discrete “biotypes” that differentially involve sensory processing impairments and are differentially distributed across inpatient and outpatient treatment settings^13,14^. Based upon this, as well as our prior observations of heterogeneity of EAP deficit across samples, we hypothesized that EAP deficits in schizophrenia would be multimodally distributed, and thus would index pathophysiologically distinct patient groups within the larger clinical syndrome. Furthermore, we hypothesized that EAP intact and impaired groups would differ in functional outcome.

In addition, we wished to test potential pathophysiological mechanisms underlying the dichotomy between EAP impaired and intact patient groups. In general, impairment in the ability to match tones following brief delay in the presence of preserved audiometric thresholds (i.e., ability to detect presence/absence of tones) reflects dysfunction in the interaction between auditory thalamus (i.e., medial geniculate nucleus: MGN) and early auditory (EA) cortex, including primary auditory cortex (A1)^12,15^.

By contrast, lower level pathology (e.g., cochlea, brainstem) would result in reduced audiometric thresholds, whereas impairments in higher order regions (e.g., frontal cortex) would increase susceptibility to distraction, but would not affect tone-matching thresholds in the absence of distractors (reviewed in ref. ^7^). Proposed localization of the EAP deficits in SZ to MGN/EAC is supported as well by our recent demonstration of hierarchically organized dysfunction within the EAC in SZ^6^, as well as histological evidence of reduced presynaptic innervation and pyramidal neuron dendritic size^16^ with primary auditory cortex in SZ^7^.

In neurophysiological studies, we have previously observed that deficits in MMN generation in SZ reflect impaired resting-state functional connectivity during fMRI (rsFC-fMRI) scans between MGN and EA^17^. Here, we investigated the degree to which disturbed MGN-EA connectivity affects behavioral TMT ability. Resting-state fMRI data were available from a large subset of subjects (*n* = 78 SZ/93 HC), permitting correlational studies between EAP deficits and underlying rsFC changes. We hypothesized that EAP- impaired, but not intact, patients would show significant reductions in rsFC between MGN and EA regions, and that such deficits would correlate with severity of TMT deficit across groups.

Finally, we investigated TMT ability in an sample of young individuals at symptomatic clinical high risk (CHR) for psychosis and age-matched controls to estimate EAP impairments from a neurodevelopmental perspective. The ontogeny of EAP dysfunction in SZ remains relatively unknown. We have recently observed that deficits in visual sensory processing, especially impaired motion detection, may predate illness onset in SZ^18^. Here we investigated the degree to EAP deficits may precede illness onset as well.

## Methods

### Participants

Participants included 310 individuals with a SCID-DSM-IV^19^ diagnosis of SZ or schizoaffective (SZaff) disorder compared with 219 representative healthy controls (HC), and 24 young individuals with a SIPS/SOPS^20^ diagnosis of CHR characterized by attenuated positive symptoms (age = 21.6 ± 4.5 years) compared with a subset of 24 age-matched HC (age = 19.8 ± 1.6 years) from a larger sample of 67 young HC (age = 19.2 ± 5.6 years). Patients were recruited between 2010 and 2018 from inpatient (*N* = 145) and outpatient (*n* = 165) settings associated with both Columbia University Medical Center (CUMC) and Nathan Kline Institute (NKI). Some subjects performed the task more than once, yielding a total of 448 SZ and 295 HC sessions pooled across studies^10,21–26^ (Table 1). All participants had no substance abuse in the past month and no dependence within the past 6 months (DSM-IV). All denied a history of head injury with loss of consciousness, or other neurological disorders. All studies were approved by the ethics committees of NKI and CUMC. All participants provided written, informed consent.

**Table 1:** Socio-demographic, psychiatric history/comorbidity and medication data across groups

Variable	Group			Statistics			
	Control (*n* = 219)	Schizophrenia (*n* = 310)		SZ-EAP+ vs. SZ-EAP−			
		SZ-EAP+ (*n* = 134)	SZ-EAP− (*n* = 176)	Test	Stat	*p*-value	Effect size
*Demographics*		*n* = 134	*n* = 176				
Age (years)	34.5 ± 9.8	38.4 ± 11.5	42.0 ± 11.0	*z*	2.82	<0.05	0.32
Sex (% Female)	23.0%	23.6%	26.4%	*χ* ^2^	2.26	–	
Diagnosis (SZ/SZaff)	–	103/31 (23.1% SZaff)	141/35 (19.9% SZaff)	*χ* ^2^	0.49	–	
Ethnicity (%)							
Hispanic/Latinos	5.8%	22.4%	15.3%	*χ* ^2^	2.52	–	
White	42.0%	35.1%	16.5%	*χ* ^2^	14.22	<0.0005	0.44
Black/African-American	39.2%	30.6%	59.1%	*χ* ^2^	24.28	<0.0001	0.58
Asian	6.5%	2.2%	1.5%	*χ* ^2^	0.11	–	
Other	6.5%	10.4%	6.8%	*χ* ^2^	0.62	–	
Hand preference (%right)	84.3%	89.0%	89.8%	*χ* ^2^	>0.01	–	
IQ	106.3 ± 8.5	96.2 ± 11.5	88.9 ± 10.3	*z*	5.79	<0.0001	0.67
Highest grade achieved	19.9 ± 11.6	16.7 ± 8.9	13.3 ± 7.2	*z*	3.61	<0.0005	0.51
Participant SES	41.9 ± 11.7	28.5 ± 11.0	26.0 ± 10.9	*z*	1.99	–	
Parents SES	44.3 ± 13.2	42.5 ± 16.1	39.4 ± 19.4	*z*	1.53	–	
*Psychiatric history*							
Age at first hospitalization	–	22.9 ± 8.8 (*n* = 47)	22.0 ± 7.9 (*n* = 85)	*z*	0.58	–	
Illness duration	–	13.3 ± 8.5 (*n* = 44)	17.0 ± 9.9 (*n* = 77)	*z*	2.17	<0.05	0.40
*Comorbidity*		*n* = 134	*n* = 176				
Personality disorder	–	13.4%	5.7%	*χ* ^2^	5.56	<0.05	0.27
Post-Traumatic stress disorder	–	23.1%	5.7%	*χ* ^2^	20.19	<0.0001	0.53
Major depression	–	50.0%	15.3%	*χ* ^2^	43.25	<0.0001	0.81
Anxiety disorder	–	0.0%	1.7%	*χ* ^2^	0.50	–	
Substance abuse	–	50.7%	18.2%	*χ* ^2^	36.92	<0.0001	0.74
Family history of psychosis (%)	–	48.1%	54.8%	*χ* ^2^	0.59	–	
*Medication*		*n* = 82	*n* = 109				
Chlorpromazine equivalent	–	718.9 ± 918.5	854.6 ± 826.7	*z*	2.36	< 0.05	0.35
Atypical (%)	–	88.6%	87.8%	*χ* ^2^	0.03	–	
Anticholinergics	–	31.6%	30.6%	*χ* ^2^	0.02	–	

Data are presented as mean ± SD

### Assessments

Estimated premorbid IQ was assessed using the Quick IQ test^27^. Neurocognitive function was assessed using Modules 1–6 (Processing Speed, Attention/Vigilance, Working Memory, Verbal Learning, Visual Learning, and Reasoning/Problem Solving) of the MCCB^2^. The primary dependent measures were T-scores.

EAP integrity was assessed using a simple tone-matching paradigm (TMT) as described previously^5,6,25^. Briefly, this task presents participants 130 pairs of 100-ms pure tones in series, with 500-ms intertone interval. Within each pair, tones are either identical or differ in frequency by specified amounts in each block (Δ2.5%, Δ5%, Δ10%, Δ20%, or Δ50%). Tones are derived from three reference frequencies (500, 1000, and 2000 Hz) to avoid learning effects. Participants were asked to assess if the tones were identical or different by forced choice (2-button) press (i.e., 50% chance performance). Stimuli are presented through headphones at a comfortable listening level. The test takes ~10 min to complete. Participant performance across the 5Δ levels was averaged and this score was used for analysis. With the exception of measures for stability of TMT over time, only baseline TMT measurement was used for all of the analyses presented across the paper.

Auditory-related social cognition was assessed using the auditory emotion recognition (AER) and sarcasm “attitudinal prosody” tests as previously described^28,29^. For these tests, the primary dependent measures were percent of correct responses.

The Positive and Negative Symptoms Scale (PANSS) was administered to patients by trained raters, with inter-rater reliability of ≥0.8. The total score was divided into separate positive, negative, cognitive, excitement/hostility, and anxiety/depression factors^30^. Functional capacity was measured with the UCSD Performance-based Skills Assessment (UPSA) task^31^.

### rsFC-fMRI analyses

rsFC-fMRI analyses were conducted in 78 SZ and 93 HC (NKI: *n* = 82, 1.5T-fMRI CUMC: *n* = 89, 3T-fMRI) using previously published methods (see Supplementary Methods 1 for details). The site of acquisition (NKI/CUMC) was used as covariate for rsFC-fMRI analyses.

Between-group differences in functional connectivity were assessed using seed-based voxel-wise analyses with bilateral seeds which were delineated in MGN as defined in the Talairach’s atlas^32^ and EA and associative auditory (AA) using regions as defined by Glasser and colleagues^33^. Correlations were Fisher z-transformed. Follow-up analyses investigated parcelwise correlations within significant regions.

### Statistical analyses

The groups were compared using Mann–Whitney tests for continuous variables, and likelihood ratio (LR) *χ*² tests for categorical data. The statistical distribution of TMT deficits is assessed using a Gaussian finite mixture model. The Bayesian information criterion (BIC) was used to select preferred models (with lowest BIC preferred), while a likelihood ratio *χ*² is used to determine differences between models^34^. As the LR model can be anti-conservative for evaluating significance, we applied parametric bootstrapping to significant *P*-values^35^.

Stability of TMT measures over time was calculated using intraclass correlation coefficients (ICC-absolute agreement).

Profiles across TMT performance, symptom factors, neurocognitive domains, and rsFC-fMRI patterns were assessed using repeated measures rmANOVA with Sidak post hoc contrasts between groups or principal component analysis (PCA) with varimax rotation, as appropriate. Effects of potential confounds (i.e., ethnicity^36^, premorbid IQ, education, medication dose, site of MRI acquisition) were assessed using ANCOVA. Relationship to functional outcome was assessed using multiple regression and mediation analyses.

Correlational analyses for individual potential predictors were conducted using Spearman non-parametric testing (*r*_s_). Relative contribution of independent predictors was assessed using multiple linear regression and consideration of partial correlation (*r*_p_) coefficients.

All statistics are two-tailed with pre-designated *α*-level of significance of *P* < 0.05. Corrections for multiple comparisons were applied, as indicated. Values in text represent mean ± standard deviation unless otherwise specified. Data were analyzed with R software (“mclust” package for gaussian mixture modeling) and SPSS version 22.

## Results

### Tone-matching performance

**Fig. 1:**
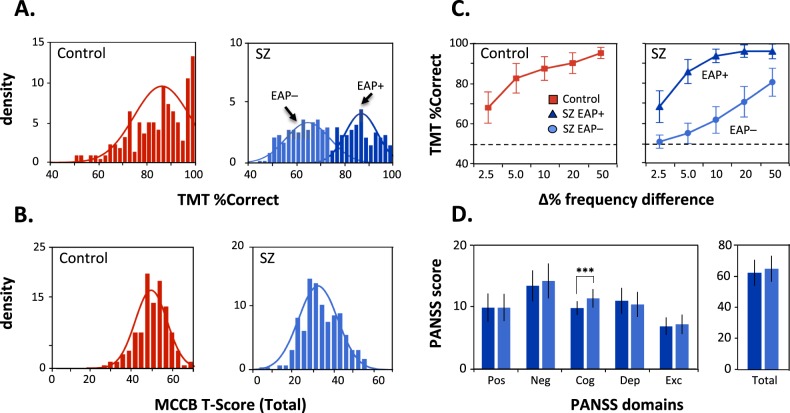
Profiles across TMT performance, neurocognitive domains and symptom factors. **a** Density histograms of TMT percent correct responses showing unimodal distribution for the Control group only. For the SZ group, the bimodal distribution had a substantially lower BIC than a unimodal model, and the bimodal/unimodal likelihood ratio was highly significant (*χ*² = 22.23, *P* < 0.0001). **b** Density histograms of MCCB total T-scores (addition of six domains) showing unimodal distribution for both Controls and SZ groups. **c** Line plots of mean percentage correct of tone-matching task for each level of frequency difference. **d** Bargraphs (mean + /−SD) of scores for PANSS domains between SZ groups. Pos positive, Neg negative, Cog cognitive, Dep depression, Exc excitement. ***P < 0.0001

As expected, SZ patients showed a highly significant, large effect-size deficit in the tone-matching task (TMT: *z* = −8.67*, P* < 0.00001, *d* = 0.98) vs. HCs (Table 2). In HCs, TMT scores did not deviate significantly from a unimodal distribution (mean = 84.9 ± 9.6% correct), which had a substantially lower BIC than a bimodal model (likelihood ratio (LR): *χ*² = 18.4, *P* < 0.001). In the SZ group, a bimodal distribution had a substantially lower BIC than a unimodal model (LR: *χ*² = 22.2, *P* = 0.0002; parametric bootstrap: LR test statistic = 44.5, *P* = 0.001) (Fig. 1a). By contrast, MCCB scores did not deviate significantly from a unimodal distribution in either SZ or HC (Fig. 1b).

**Table 2:** Neurocognition, auditory-related social cognition, and function measures across groups

Variable	Group			Statistics		
	Control	Schizophrenia		SZ-EAP + vs. SZ-EAP−		
		SZ-EAP +	SZ-EAP−	*z* stat	*P*-value	effect size
*Neurocognition (MCCB)*						
Processing speed	49.7 ± 7.4 (*n* = 83)	34.7 ± 12.9 (*n* = 108)	23.7 ± 11.6 (*n* = 136)	6.92	<0.0001	0.99
Attention/vigilance	47.6 ± 8.8 (*n* = 135)	38.2 ± 12.9 (*n* = 117)	27.8 ± 11.5 (*n* = 150)	6.85	<0.0001	0.92
Working memory	49.1 ± 9.3 (*n* = 86)	38.1 ± 12.5 (*n* = 108)	27.1 ± 11.6 (*n* = 135)	7.04	<0.0001	1.01
Verbal learning	45.6 ± 6.9 (*n* = 98)	37.5 ± 8.5 (*n* = 108)	31.9 ± 7.8 (*n* = 136)	5.30	<0.0001	0.72
Visual learning	44.6 ± 8.6 (*n* = 96)	36.6 ± 13.4 (*n* = 110)	29.2 ± 12.4 (*n* = 146)	4.52	<0.0001	0.59
Reasoning/problem Solving	46.0 ± 9.4 (*n* = 94)	40.3 ± 10.4 (*n* = 108)	35.4 ± 8.7 (*n* = 136)	3.93	=0.0001	0.52
*Auditory emotion* (%)	*n* = 189	*n* = 46	*n* = 37			
Total	72.7 ± 17.1	59.7 ± 11.2	50.3 ± 12.9	3.95	<0.0001	0.88
Pitch	58.9 ± 18.4	54.6 ± 16.8	40.6 ± 16.4	4.13	<0.0001	0.92
Intensity	50.4 ± 18.7	50.3 ± 19.0	40.9 ± 19.1	2.41	<0.05	0.51
Happy	78.5 ± 27.8	38.9 ± 21.9	30.2 ± 20.5	2.01	<0.05	0.41
Sad	85.2 ± 15.2	74.1 ± 22.0	62.4 ± 23.9	2.48	<0.05	0.52
Anger	87.5 ± 14.0	73.5 ± 17.1	60.4 ± 23.4	3.08	<0.005	0.66
Fear	20.9 ± 21.1	46.9 ± 16.9	31.2 ± 17.3	4.49	<0.0001	1.02
Neutral	82.9 ± 21.8	72.4 ± 23.7	62.8 ± 28.0	1.79	–	
*Sarcasm* (%)	*n* = 54	*n* = 74	*n* = 89			
Total	83.5 ± 12.2	73.2 ± 14.9	64.6 ± 12.9	3.42	<0.0001	0.65
Sensitivity A′	90.4 ± 4.2	83.8 ± 1.3	76.7 ± 6.1	9.03	<0.0001	2.81
Bias B″	43.5 ± 3.0	42.3 ± 0.8	38.9 ± 2.1	1.54	–	
*Function*		*n* = 106	*n* = 132			
UPSA total		69.3 ± 16.4	58.4 ± 18.2	4.85	<0.0001	0.66

*MCCB* Matrics Cognitive Consensus Battery, *UPSA* UCSD Performance-based Skills Assessment

Data are presented as mean ± SD

Within SZ, two clusters were identified: SZ-EAP + (*n* = 142, weight = 42.2%, mean = 87.8 ± 6.8% correct) and SZ-EAP− (*n* = 168, weight = 57.8%, mean = 64.1 ± 8.4% correct). Mean score in the SZ-EAP + group was not significantly different from that of controls (*z* = −1.41, *P* = 0.16). By contrast, scores within the SZ-EAP− group were significantly reduced from both controls (*z* = −14.5, *P* < 0.0001) and SZ-EAP + (*z* = −15.1, *P* < 0.0001). The threshold between clusters was 77.7% correct tone-matching responses. When this cut-off was applied across groups, 166 of 310 (53.5%) of SZ patients scored below this cut-off vs. only 46 of 219 (21.7%) of controls (LR: *χ*² = 59.0, *P* < 0.00001) (Fig. 1c).

The percentage of EAP− subjects among patients with SZ (112/244, 45.9%) did not differ statistically (*χ*² = 0.14, *P* = 0.70) from the percentage among patients with SZaff disorder (32/66, 48.5%). The percentage of EAP− subjects among patients from inpatient sites (94/145, 64.8%) was significantly higher (*χ*² = 7.20, *P* = 0.007) than the percentage of EAP + subjects from outpatient sites (82/165, 49.7%). Within both EAP− and EAP+ groups, there was no significance difference in TMT scores between in- and outpatients.

### Demographic contributions

SZ-EAP− were significantly older than SZ-EAP+ individuals at the time of testing (Table 1). Nevertheless, differences remained strongly significant even following covariation for age (*F*_(1,302)_ = 853.1, *P* < 0.00001), whereas the relationship between age and TMT was not significant (*F*_(1,302)_ = 2.70, *P* = 0.10). The two groups also differed significantly in race, with greater relative prevalence of SZ-EAP− vs. SZ-EAP+ among Black/African-American ethnicity vs. white ethnicity individuals. Nevertheless, the SZ-EAP+ vs. SZ-EAP− difference remained highly significant even when ethnicity was included as a factor (*F*_(1,293)_ = 205.2, *P* < 0.00001).

The SZ-EAP− and SZ-EAP+ groups also differed in estimated premorbid IQ. Although a significant association between premorbid IQ and TMT score was observed (*F*_(1,236)_ = 14.6, *P* = 0.0002), the difference in TMT score between SZ-EAP− and SZ-EAP+ groups remained strongly significant even following covariation for premorbid IQ (*F*_(1,236)_ = 552.6, *P* < 0.00001).

Finally, the SZ-EAP+ and SZ-EAP− groups differed in educational level such that SZ-EAP− were significantly less likely than SZ-EAP+ to have pursued education beyond high school (25.5 vs. 50.9%, *χ*² = 18.3, *P* < 0.00001). Medication dose, as expressed in CPZ equivalents was significantly higher in SZ-EAP− than SZ-EAP+ subjects. Nevertheless, the between-group difference remained highly significant even when educational level (*F*_(1,271)_ = 735.0, *P* < 0.00001) or CPZ equivalents (*F*_(1,188)_ = 505.2, *P* < 0.00001) were included as covariates.

### Symptom and neurocognitive differences between groups

Total PANSS scores were not significantly different between SZ groups (Table 2). However, the 5-factor symptom profile^30^ differed significantly, such that SZ-EAP− subjects showing significantly greater levels of cognitive symptoms than SZ-EAP +, particularly involving difficulty in abstract thinking, disorientation and poor attention. When these variables were entered into a discriminant function against illness type, they correctly identified 65.2% of subjects (*χ*² = 26.2, df = 3, *P* < 0.00001).

SZ-EAP− subjects also showed an overall reduction in MCCB scores (*z* = 5.42, *P* < 0.00001) relative to SZ-EAP +. In rmANOVA, the group × domain interaction was also highly significant (*F*_(5,235)_ = 31.6, *P* < 0.00001), indicating differential impairment across domains. When MCCB domains were entered into a stepwise discriminant function analysis, domains 1–3 [Processing Speed, Attention/Vigilance, Working Memory] all emerged as significant factors (all *P* < 0.0001), whereas other domains did not enter.

Consistent with this, SZ-EAP− subjects also showed significantly greater mean difference between MCCB domains 1–3 [Processing Speed, Attention/Vigilance, Working Memory] vs. between domains 4–6 [Verbal Learning, Visual Learning, Reasoning/Problem Solving] (6.0 ± 7.4 vs. 1.1 ± 7.3, *z* = 4.70, *P* < 0.00001) that correctly identified 61.1% of SZ-EAP− vs. SZ-EAP + patients (*χ*² = 19.0, *P* < 0.0001).

The differential impairment in processing speed that we observed in the SZ-EAP− vs. SZ-EAP+ subjects also correlated with medication dosage across SZ subjects (*r*_s_ = 0.22, *P* = 0.009), along with TMT performance (*r*_s_ = −0.38, *P* < 0.0001) and estimated premorbid IQ (*r*_s_ = −0.31, *P* < 0.0001). When entered into a simultaneous multiple regression, only TMT performance emerged as a significant correlate (*r*_p_ = −0.26, *P* = 0.012), whereas effects of premorbid IQ (*r*_p_ = −0.13, *P* = 0.23) and medication dosage (*r*_p_ = 0.29, *P* = 0.14) were no longer significant.

### Contribution to auditory-related social cognition deficits

**Fig. 2:**
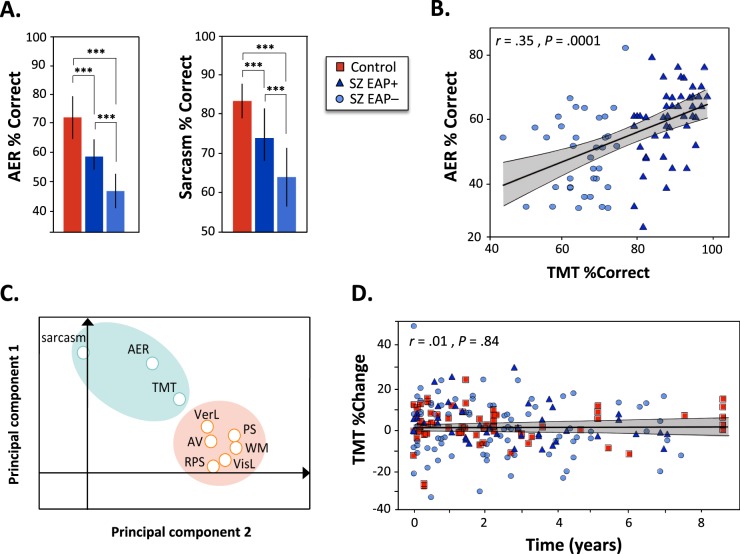
Auditory-related social cognition performances, relationships with TMT and neurocognition, and longitudinal stability of TMT. **a** Bargraph (mean + /−SD) of performance on auditory emotion recognition (AER) and sarcasm detection tasks. *Y*-axis is calibrated to start from chance performance. **b** Scatter plot of total score for percentage correct on tone-matching task versus performance on auditory emotion recognition. Gray shaded area = IC 95%. **c** Principal Component Analysis biplot showing two clusters: MCCB domains T-scores (orange) and tone-matching task (TMT), auditory emotion recognition (AER) and sarcasm percent correct (green). The two principal components captured 63.6% of the data variability. PS processing speed, AV attention/vigilence, WM working memory, VerL verbal learning, VisL visual learning, RPS reasoning/problem solving. **d** Scatter plot illustrating % change in tone-matching task scores across time. Gray shaded area = IC 99%. ICC values were extremely high for SZ subjects who participated in 2- (*n* = 60, ICC = 0.95) or 3- (*n* = 31, ICC = 0.99) sessions, as well as for controls (2-session: *n* = 45, ICC = 0.90; 3-session: *n* = 20, ICC = 0.96). ***P < 0.0001

AER (*n* = 97) and sarcasm (*n* = 123) measures were available for a subset of SZ subjects. Samples of both inpatients (AER: *n* = 46, sarcasm: *n* = 70) and outpatients (AER: *n* = 51, sarcasm: *n* = 53) of comparable sizes were tested. Both sets of measures differed significantly between SZ-EAP− and SZ-EAP + subjects (Fig. 2a). In discriminant function analysis, 71.1% of EAP− vs. EAP + subjects could be identified using AER (*χ*² = 11.8, *P* = 0.001). Similarly, sarcasm detection significantly discriminated between groups (*χ*² = 8.43, *P* = 0.004). Both AER (Fig. 2b, *r*_s_ = 0.34, *P* < 0.005) and sarcasm detection (*r*_s_ = 0.35, *P* < 0.0001) correlated significantly with TMT across EAP− and EAP + groups.

In order to evaluate the relationship between EAP deficits (TMT), EAP-related social cognition deficits (AER, sarcasm) and other aspects of neurocognitive impairment (MCCB), we conducted a PCA across measures. MCCB domains 1–6 loaded onto a single factor that accounted for 38.5% of the variance in scores within the SZ group. AER and sarcasm measures loaded onto a second factor that accounted for an additional 20.8% of the variance. TMT deficits loaded primarily into factor 2 (loading weight = 0.52), but contributed as well to factor 1 (loading weight = 0.49) (Fig. 2c).

### Contributions to functional capacity

EAP− and EAP + subjects differed significantly in functional capacity, as assessed using the UPSA (Table 2). In order to evaluate the relative effects, TMT and MCCB total scores were entered into a simultaneous regression. TMT (*r*_p_ = 0.18, *P* = 0.004) contributed to reduced UPSA scores over and above the contribution of MCCB dysfunction (*r*_p_ = 0.37, *P* ≤ 0.0001).

A follow-up regression evaluated contributions of individual MCCB domains, relative to TMT. In this analysis, TMT again contributed significantly to UPSA (*r* = 0.42, *P* < 0.0001), over and above effects of working memory (*r*_p_ = 0.31, *P* < 0.0001) and verbal learning (*r*_p_ = 0.18, *P* = 0.005).

Given the joint effects of TMT and working memory on functional capacity, we conducted a mediation analysis to investigate the degree to which working memory deficits mediate the TMT effect. TMT had a highly significant total effect (*z* = 5.31, *P* < 0.0001) on UPSA along with a significant indirect effect (*z* = 3.33, *P* < 0.0001). Nevertheless, the direct effect of TMT on UPSA also reached significance (*z* = 2.62, *P* = 0.009), suggesting effects of TMT on functional capacity independent of effects on neurocognition.

### Longitudinal stability of TMT

A subset of subjects participated in multiple TMT sessions as part of repeated study participation, with intervals of up to ~9 years. ICC values were extremely high for SZ and controls subjects who participated in 2- or 3-sessions (Fig. 2d). Moreover, when percent change scores were assessed by subject over time the regression line across groups was flat, with mean % variability across all time points of 2.72 ± 11.0%. In addition, when baseline TMT score was entered in a covariance analysis with percent change scores and group as factors, we observed that higher baseline scores significantly predicted higher stability over time across groups (*F*_(1,0.30)_ = 27.9, *P* < 0.00001).

### Pathophysiological mechanisms

**Fig. 3:**
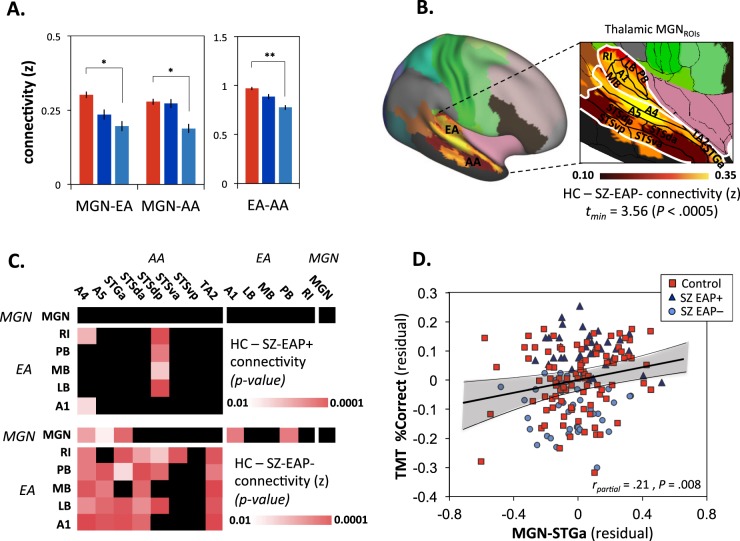
Resting-state functional connectivity MRI patterns and relationships with TMT. **a** Bargraph (mean + /− SD) of resting-state functional connecting *z*-scores between Glasser’s regions across groups. *P < 0.05; **P < 0.005. **b** Voxel-wise comparisons between controls and SZ-EAP− with bilateral thalamic (MGN) ROIs based on Glasser’s regions for auditory pathway. **c** Statistical Heatmaps representing comparisons for resting-state functional connecting *z*-scores per bilateral parcel for Controls vs. SZ-EAP− and Controls vs. SZ-EAP +. **d** Scatter plot of total percent correct on tone-matching performance versus rsFC-MRI between MGN and STGa, which belongs to AA. Partial *r* was computed across two sites (outpatient and inpatient) and two groups (Controls and SZ). *Parcellations*: AA = associative auditory: A4 = Brodmann area A4, A5 = Brodmann area A5, STGa = anterior superior temporal gyrus, STSda = dorsoanterior superior temporal sulcus, STSdp = dorsoposterior superior temporal sulcus, STSva = ventroanterior superior temporal sulcus, STSvp = dorsoposterior superior temporal sulcus, TA2 = anterosuperior temporal area. EA = early auditory: A1 = primary auditory, LB = lateral belt, MB = medial belt, PB = parabelt, RI = retro-insula. MGN = thalamic medial geniculate nuclei

MRI data were available from a subset of subjects (*n* = 93 HC, 38 SZ-EAP+, 40 SZ-EAP–). In order to assess underlying neural mechanisms, we evaluated pairwise rsFC between bilateral subcortical (MGN) and cortical (EA, AA) auditory regions across groups, controlling for site of MRI acquisition. The main effect of group was highly significant across all pairwise comparisons MGN-EA, MGN-AA, EA-AA) (*F*_(1,167)_ = 6.99, *P* = 0.001), with significant reduction in bilateral rsFC in SZ-EAP− relative to HC (Sidak post hoc *P* = 0.001) (Fig. 3b). By contrast, no significant difference was observed between SZ-EAP + and either HC (post hoc *P* = 0.40) or SZ-EAP− (Sidak post hoc *P* = 0.20) subjects. The group × region interaction was not significant (*F*_(4,334)_ = 1.55, *P* = 0.19), reflecting similar magnitude reduction across all pairwise comparisons (Fig. 3a). Between-group results remained significant even following covariation for site of data acquisition (*F*_(2,165)_ = 4.79, *P* = 0.009). The site × region (*F*_(2,330)_ = 1.28, *P* = 0.28) and group × site × region (*F*_(4,330)_ = 0.995, *P* = 0.41) interactions were non-significant.

When data were mapped onto individual parcels, significant reductions were observed between MGN and both EA and AA in SZ-EAP− subjects, as well as reductions between EA and AA, particularly involving AA areas A4, A5, STGa, and TA2, which are immediately adjacent to EA (Sidak post hoc *P* < 0.01 with covariation for site). For SZ-EAP+ subjects, significant reductions were observed only in EA-AA connectivity (Fig. 3c). These differences were predominant in the right hemisphere when MGN was used as the seed region, and in the left hemisphere when EA and AA were used as seeds (Supplementary Fig. 1). Strongest correlation with TMT across groups was observed between MGN-anterior superior temporal gyrus (STGa), with significant partial *r* controlling for group and site (Fig. 3d).

### Developmental trajectory

As opposed to deficits in SZ, no significant differences for TMT were observed between CHR and age-matched HC (CHR = 91.9 ± 8.1%, HC = 89.8 ± 10.0% correct, *z* = −0.83, *P* = 0.40) (see Supplementary Table 1 for details). In the CHR group, 22 individuals were EAP+ (91.7%) and 2 were EAP− (8.3%) according to TMT performances.

In the larger group of young HC (*n* = 67), TMT performance increased linearly with age over the years (age range = 7–27 years, *r* = 0.36, *P* = 0.003), reaching the threshold that distinguishes EAP+ from EAP− (77.7% correct) between ~7 and 12 years of age (Supplementary Fig. 2).

## Discussion

### Tone-matching indexes dichotomous groups

Since the term “schizophrenia” was first introduced in the early 1900’s, it has been considered to refer to a group of disorders rather than a single nosological entity^1^. Nevertheless, attempts to date to identify discrete pathophysiological subgroups have largely failed. In parallel, there has been increasing appreciation over recent years that sensory processing measures, especially those involving EAP (e.g., tone-matching) may segregate between SZ patients who require continued supervised care vs. those capable of independent living and outpatient supervision^6,12^.

Here, using a large sample of SZ and SZaff disorder subjects recruited from both inpatient and outpatient settings, we provide the first demonstration that the EAP deficits, as indexed by TMT, are dichotomously distributed across subjects, with one group showing entirely unimpaired EAP performance, and the second group showing an extremely large impairment relative to both controls (*d* = 2.1) and EAP+ patients (*d* = 3.4). Furthermore, we showed that TMT performance is highly reliable measure across time in individuals with established SZ, although the course of the deficits at early stages of the illness (e.g., <3 years) remains to be determined.

Consistent with the present results, auditory-related neurophysiological measures such as auditory N1 gating, P3 and MMN have also been proposed to differentiate between subtypes^10,13^. However, such measures are too complex for implementation in routine clinical practice, but might be used in research settings. In contrast to the EAP measures, neither symptoms nor MCCB scores were dichotomously distributed across our SZ sample. These findings are consistent with large-scale validation trials of both sets of measures^2,37^, and suggest that deficits in higher order cognition and symptoms are multi-determined and less specific for SZ subtypes than EAP measures.

As predicted^12^, the EAP− group predominated among samples drawn from inpatient sites whereas the EAP+ group predominated from those drawn from outpatient sites. The EAP− and EAP+ groups, moreover, differed in terms of symptoms, neuropsychological profiles and brain activation patterns. In symptoms, the two groups did not differ in severity of either positive or negative symptoms. However, EAP− patients showed higher levels of cognitive symptoms and especially of difficulty in abstract thinking, disorientation and poor attention.

In neurocognition, SZ-EAP− subjects showed a greater deficit overall than did SZ-EAP+, as well as a markedly uneven profile across domains. Although the basis for the differential cognitive impairment in SZ-EAP− vs. SZ-EAP+ groups requires further investigation, one potential mechanism is differential thalamic involvement. Thalamic dysfunction is increasingly recognized as a major contributor to cognitive impairment in SZ^38,39^. Further studies are needed to determine the degree to which the impaired MGN/AA connectivity that we observed in the SZ-EAP− group extends to other thalamic regions as well.

Both TMT and working memory deficits contributed in parallel to impairments in functional capacity as measured by the UPSA. Although functional outcome was not measured directly in the present study, prior studies have shown strong relationships between UPSA and real-world functioning^30^, consistent with previously reported associations between EAP deficits and functional outcome^7,8,11,40^.

### Relationship to symptoms and neurocognition

Our findings may also shed light on heterogeneity of neuropsychological findings across studies in SZ. Thus, in studies of MCCB involving primarily outpatients, a consistent reduction of performance is observed across all domains^41^, similar to what we observe here in the SZ-EAP+ group. By contrast, other groups working from combined inpatient and outpatient samples have suggested preferential impairment in processing speed relative to other neurocognitive domains, similar to what we observed in SZ-EAP− subjects, but with significant heterogeneity of deficit across samples^42^.

Such studies stressed the contributions of both medication dose and overall cognition as predictors of differential processing speed across cohorts. In the present study, we observed that the differential reduction in processing speed was related to tone-matching, estimated premorbid IQ and medication dosage independently. However, when put into a multiple regression, only the effects of TMT performance remained significant.

Similarly, correlations between medication dosage and PANSS cognitive symptoms were no longer significant once TMT performance was considered. By contrast, TMT performance remained significantly correlated with symptoms. The importance of cognitive symptoms to functional outcome has been noted previously^43^. The present study suggests that rather than directly driving outcome, they may index a poor outcome form of the disorder.

### Relationship to pathophysiological mechanisms

rsFC-fMRI analyses showed significant reduction in SZ-EAP− relative to HC between major auditory areas (EA, AA, MGN), while these differences were not observed in SZ-EAP+ relative to HC subjects. Conversely, relative to SZ-EAP+, significant reductions were observed in SZ-EAP− involving MGN to A1, A4, A5, the parabelt and the anterior superior temporal gyrus (STGa). This suggests that SZ-EAP− may display impairments at a very early, pre-cortical level along the auditory pathway.

In addition, a significant correlation with TMT across groups was observed between MGN and the STGa. This is supported by previous studies that demonstrated both tone-matching and STGa involvement in theory of mind and emotion recognition processes and the prevalence of their impairments among SZ individuals^6,44,45^.

Thus, our neuroimaging findings provide further biological evidence for the distinction of two discrete subgroups of SZ based on auditory processing abilities.

### Implication for early identification and treatment

In our CHR sample, only 8.3% of subjects qualified as EAP−, suggesting either that deficits develop during the initial years of the illness (e.g., ref. ^46^) or that individuals with premorbid EAP deficits are not being captured by current CHR criteria and recruitment approaches. EAP accuracy appears to increase linearly throughout neurodevelopment^47^. Thus, healthy volunteers reach the threshold that separates EAP+ from EAP− (77.7% correct responses) starting at ages ~7–12 years of age. Thus, deficits could be attributed to either a developmental lag beginning at ~age 12 (e.g., ref. ^48^) or neurodegeneration from a higher level of function (e.g., ref. ^46^). Future longitudinal studies in early stage patients are needed to distinguish these possibilities.

The difference in disease etiology between SZ-EAP− and SZ-EAP+ groups may also necessitate different clinical strategies. For example, in one study where EAP status was assessed before entering subjects into cognitive remediation, only SZ-EAP− subjects benefited significantly from auditory-based remediation. In these subjects, both EAP and verbal learning improved^49^. In parallel, in a recent study of transcranial direct current stimulation (tDCS) for auditory hallucinations, we observed significant benefit only in SZ-EAP+ patients^50^, suggesting that EAP− subjects may lack the functional substrates necessary for effective tDCS-based modulation of local connectivity.

### Limitations

One limitation to this study is that sample sizes were not sufficient to split the sample for identification and replication of the EAP subtypes. Further studies are therefore needed to cross-validate the results. The potential differential distribution across racial and ethnic categories also requires further evaluation. All patients were also receiving antipsychotic medication, with EAP− patients receiving somewhat higher doses that EAP+ (Table 1). Nevertheless, between-group differences remained strongly significant even in medication dose was considered. Most patients were also ascertained relatively long after first diagnosis. Follow-up studies specifically with first episode subjects are therefore required.

## Conclusion

In summary, clinicians are well aware that the term “schizophrenia” as presently used applies to individuals with markedly divergent clinical course. To date, no measures have been reported that allow for differentiation of dichotomous subgroups^3^. Here, we provide the first evidence that EAP, as reflected in tone-matching ability, significantly discriminates dichotomous subpopulations of individuals with SZ, suggesting discrete underlying pathophysiological mechanisms.

The EAP measure responds to an important clinical need, as it can be readily implemented within routine clinical settings, may significantly account for the heterogeneity of clinical outcomes currently observed across patients, and may be critical for pre-clinical detection and treatment selection. Finally, stratification of individuals by subtype may significantly reduce the heterogeneity of findings currently obtained across both observational and treatment outcome studies in SZ.

## Supplementary information

Supplementary Methods 1.

Supplementary Table 1.

Supplementary Figure 1.

Supplementary Figure 2.
