# Supplementary material for: Bimodal distribution of tone-matching deficits indicates discrete pathophysiological entities within the syndrome of schizophrenia
Source: Transl Psychiatry. 2019 Sep 6;9:221. doi: 10.1038/s41398-019-0557-8 (PMC6731304; doi:10.1038/s41398-019-0557-8)
Supplement: Supplementary file 1 — Supplementary Methods 1. [file 41398_2019_557_MOESM1_ESM.docx]

**Supplementary Methods 1: Acquisition and processing for rsFC-fMRI analyses.**

**Acquisition:**

rsFC-fMRI analyses were conducted in 78 SZ and 93 HC. Two distinct methods were used as participants’ scans were collected from two different sites’ databases (NKI: N = 82, CUMC: N = 89). For this reason, the site of acquisition (NKI/CUMC) was used as covariate for each analysis. Participants from CUMC were scanned with a 3T-fMRI scan and from NKI with a 1.5T-fMRI scan. On the scanner bed, participants from both sites were asked to fixate on a white dot in the centre of a black screen for a 20-to-30 minutes total duration. Potential head movements were stabilized with cushioning and MR-related sounds were minimized with earplugs.

**Processing:**

For CUMC data, resting state functional data was collected in a 3T scanner (GE-MR750) with a multiband SMS-EPI sequence (courtesy of the Center for Cognitive and Neurobiological Imaging, Stanford University, <http://cni.stanford.edu>), resolution 2mm isotropic, TR=850ms. Volume data was pre-processed with the Human Connectome Project (HCP) processing pipeline v3.4^49^. The pipeline first processed the anatomy images to create a cortical surface model for each subject aligned to the HCP surface fs_LR 32k atlas (using Glasser’s parcels^49^). Functional runs were corrected for movement and distortion, then aligned in a volume space atlas of the Montreal Neurological Institute (MNI152, 2x2x2 mm resolution, TR = 850 ms)^49,50^ and on the surface atlas created by the HCP pipeline. Additional post-processing procedures as described elsewhere^51^ were performed to minimize artifact relative to head motion, tissue signals and their derivatives, including global signal regression^51^. MR frames with a Framewise Displacement > 0.2mm were censored and replaced by interpolation. Finally, data was filtered to 0.0005-0.588 Hz. Only non-interpolated data was included in the pair-wise correlations. Each individual’s correlation matrix was Fisher-z transformed before group averaging and statistics. For both EA and AA, pairwise correlation scores were averaged across included ROIs in the HCP surface atlas (**Figure 2C**).

For NKI, data was collected in a 1.5T scanner with single-band sequences. The DPARSFA/DPABI pipeline was used^52^. Volumes were removed to result in a time series length of 145 volumes. Then, motion correction was performed and the anatomical scans were registered to the functional image. The anatomical image was segmented and a covariate analysis was performed using statistical parametric mapping priors (i.e. white matter signal, cerebrospinal-fluid signal and motion parameters). Then, images were registered to SPM’s EPI template in MNI space (3x3x3 mm resolution). Finally, the data was smoothed through a 6mm Gaussian kernel and filtered to 0.01-0.1Hz.

**References:**

49. Glasser MF, Sotiropoulos SN, Wilson JA, et al. The minimal preprocessing pipelines for the Human Connectome Project. Neuroimage 2013; 80:105-24.

50. Evans AC, Marrett S, Neelin P, et al. Anatomical mapping of functional activation in stereotactic coordinate space. Neuroimage 1992; 1(1): 43-53.

51. Power JD, Schlaggar BL, Lessov-Schlaggar CN, Petersen SE. Evidence for hubs in human functional brain networks. Neuron 2013; 79(4):798-813.

52. Yan CG, Wang XD, Zuo XN, Zang YF. DPABI: Data Processing & Analysis for (Resting-State) Brain Imaging. Neuroinformatics 2016; 14(3):339-51.
