# Supplementary material for: Bimodal distribution of tone-matching deficits indicates discrete pathophysiological entities within the syndrome of schizophrenia
Source: Transl Psychiatry. 2019 Sep 6;9:221. doi: 10.1038/s41398-019-0557-8 (PMC6731304; doi:10.1038/s41398-019-0557-8)
Supplement: Supplementary file 2 — Supplementary Table 1. [file 41398_2019_557_MOESM2_ESM.docx]

**Supplementary Table 1:**

| **Variable** | **Age-matched controls** (*N* = 24) | **CHR** (*N* = 24) |
| --- | --- | --- |
|  |  |  |
| **Demographics** |  |  |
| Age (years) | 19.8 ± 1.6 | 21.6 ± 4.5 |
| Female (%) | 45.8% | 45.8% |
| Hand preference (% right) | 95.8% | 100.0% |
| Highest grade achieved | 13.1 ± 1.6 | 13.3 ± 2.4 |
| Participant SES | 31.6 ± 11.8 | 31.2 ± 12.9 |
| Substance abuse | – | 54.2% |
| Family history of psychosis (%) | – | 58.3% |
| Chlorpromazine equivalent | – | 43.8 ± 141.2 |
| **Tone-Matching Task** (%Correct) |  |  |
| Total | 89.8 ± 10.0 | 91.9 ± 8.1 |
| 2.5% | 74.0 ± 19.0 | 79.3 ± 15.3 |
| 5% | 89.3 ± 13.0 | 92.0 ± 10.3 |
| 10% | 93.9 ± 10.0 | 94.4 ± 10.3 |
| 20% | 94.4 ± 11.0 | 94.9 ± 8.2 |
| 50% | 97.4 ± 3.0 | 98.9 ± 2.1 |

**Socio-demographic, psychiatric history/comorbidity and medication data across CHR and age-matched controls groups**.

Data is presented as mean ± SD.
