# Supplementary material for: Bimodal distribution of tone-matching deficits indicates discrete pathophysiological entities within the syndrome of schizophrenia
Source: Transl Psychiatry. 2019 Sep 6;9:221. doi: 10.1038/s41398-019-0557-8 (PMC6731304; doi:10.1038/s41398-019-0557-8)
Supplement: Supplementary file 3 — Supplementary Figure 1. [file 41398_2019_557_MOESM3_ESM.pdf]

A.

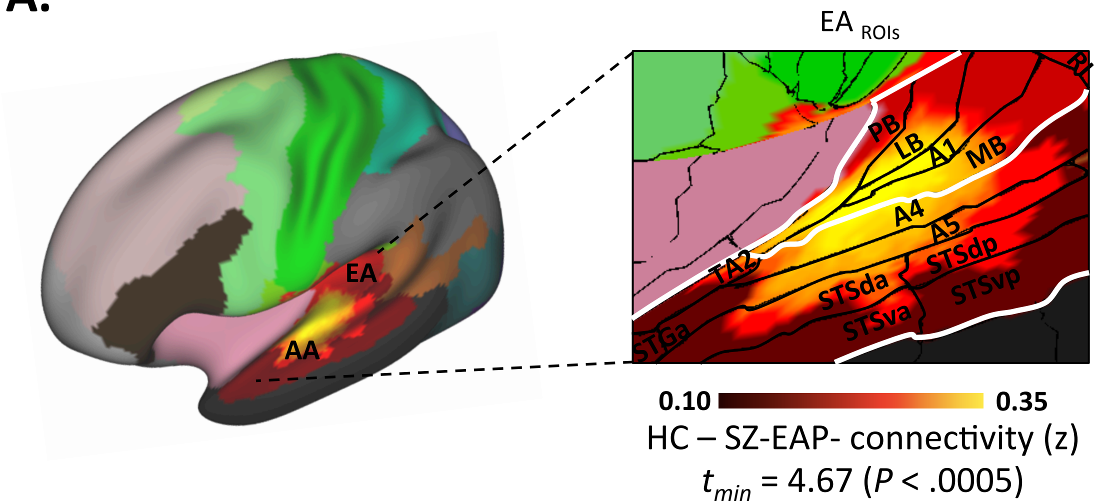

EA ROIs
EA
AA
PB
LB
A1
MB
A4
A5
TA2
STSdp
STSda
STSvp
STSva
STGa
RI
0.10
0.35
HC – SZ-EAP- connectivity (z)
t_{min} = 4.67 (P < .0005)

B.

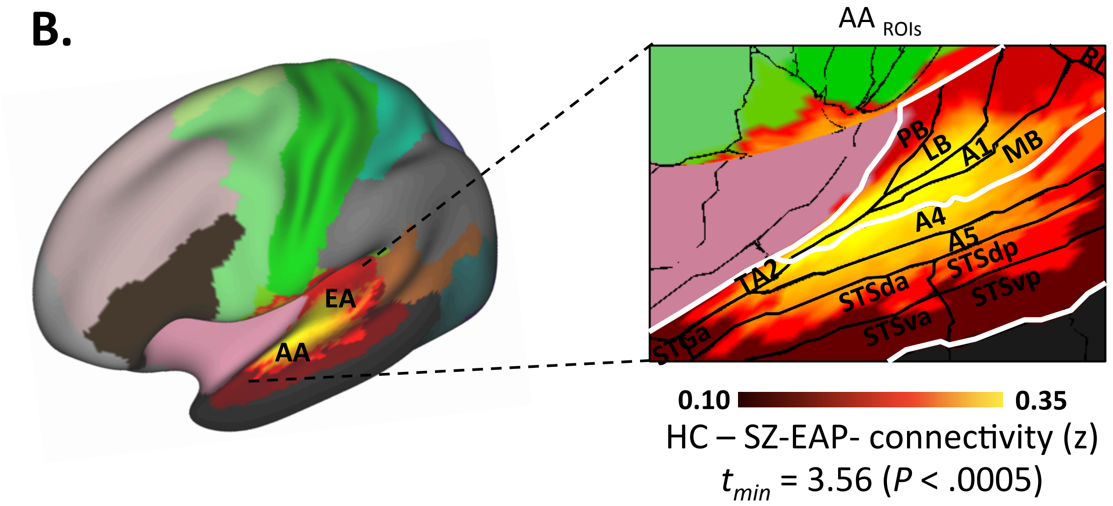

AA ROIs
EA
AA
PB
LB
A1
MB
A4
A5
TA2
STSdp
STSda
STSvp
STSva
STGa
RI
0.10
0.35
HC – SZ-EAP- connectivity (z)
t_{min} = 3.56 (P < .0005)

Supplementary Figure 1:

A. Voxel-wise comparisons between controls and SZ-EAP- with bilateral EA (Early Auditory) ROIs based on Glasser's regions for auditory pathway.

B. Voxel-wise comparisons between controls and SZ-EAP- with bilateral AA (Associative Auditory) ROIs based on Glasser's regions for auditory pathway.

Parcellations:

- AA = Associative Auditory: A4 = Brodmann area A4, A5 = Brodmann area A5, STGa = anterior superior temporal gyrus, STSda = dorsoanterior superior temporal sulcus, STSdp = dorsoposterior superior temporal sulcus, STSva = ventroanterior superior temporal sulcus, STSvp = dorsoposterior superior temporal sulcus, TA2 = anterosuperior temporal area.
- EA = Early Auditory: A1 = primary auditory, LB = lateral belt, MB = medial belt, PB = parabelt, RI = retro-insula.
