# Supplementary material for: Bimodal distribution of tone-matching deficits indicates discrete pathophysiological entities within the syndrome of schizophrenia
Source: Transl Psychiatry. 2019 Sep 6;9:221. doi: 10.1038/s41398-019-0557-8 (PMC6731304; doi:10.1038/s41398-019-0557-8)
Supplement: Supplementary file 4 — Supplementary Figure 2. [file 41398_2019_557_MOESM4_ESM.pdf]

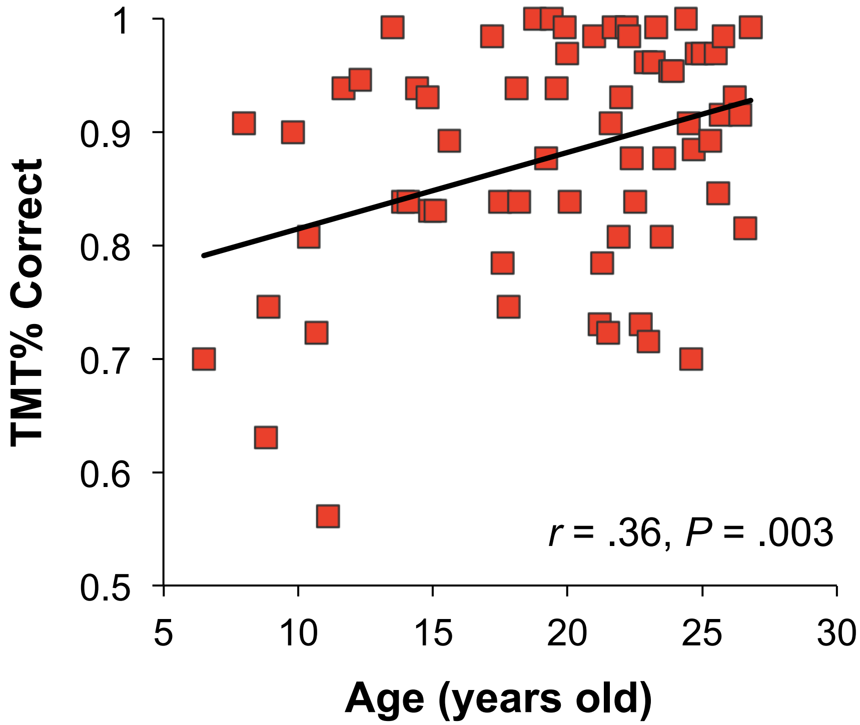

Supplementary Figure 2: Scatter plot of total score for percentage correct on tone-matching task versus age in controls.
